# How to Protect Natural Habitats of Rare Terrestrial Orchids Effectively: A Comparative Case Study of *Cypripedium calceolus* in Different Geographical Regions of Europe

**DOI:** 10.3390/plants10020404

**Published:** 2021-02-20

**Authors:** Anna Jakubska-Busse, Spyros Tsiftsis, Michał Śliwiński, Zdenka Křenová, Vladan Djordjević, Corina Steiu, Marta Kolanowska, Petr Efimov, Sebastian Hennigs, Pavel Lustyk, Karel (C.A.J.) Kreutz

**Affiliations:** 1 Department of Botany, Institute of Environmental Biology, University of Wrocław, Kanonia Street 6/8, 50-328 Wrocław, Poland; 2 Department of Forest and Natural Environment Sciences, International Hellenic University, GR-66132 Drama, Greece; stsiftsis@for.ihu.gr; 3 Lower Silesian Ecological Club, Piłsudskiego Street 74, PL-50-020 Wrocław, Poland; michal.sliwinski@o2.pl; 4 Department of Biodiversity Research, Global Change Research Institute AS CR, Bělidla 4a, 603 00 Brno, Czech Republic; zd.krenova@gmail.com (Z.K); marta.kolanowska@biol.uni.lodz.pl (M.K.); 5 Institute for Environmental Studies, Faculty of Science, Charles University, Benátská 2, CZ-12900 Prague, Czech Republic; 6 Institute of Botany and Botanical Garden, Faculty of Biology, University of Belgrade, Takovska 43, 11000 Belgrade, Serbia; vdjordjevic@bio.bg.ac.rs; 7 Association P.P.V.N.C. Excelsior, Timisoara branch, 310465 Timisoara, Romania; corinaepipactis@gmail.com; 8 Department of Geobotany and Plant Ecology, Faculty of Biology and Environmental Protection, University of Łódź, Banacha 12/16, 90-237 Łódź, Poland; 9 Komarov Botanical Institute of the Russia Academy of Sciences, 197376 Saint-Petersburg, Russia; efimov81@mail.ru; 10 Berliner Allee 191, D-13088 Berlin, Germany; info@hennigs-photography.de; 11 Nature Conservation Agency of the Czech Republic, Kaplanova 1931/1, CZ-14800 Prague, Czech Republic; pavel-lustyk@seznam.cz; 12 Department of Botany, Naturalis Biodiversity Center, 2300 RA Leiden, The Netherlands; karel.kreutz@naturalis.nl

**Keywords:** *Cypripedium calceolus*, orchids, conservation biology, threatening processes, climate changes, appropriate management

## Abstract

In this article we present and discuss the main factors that threaten natural populations of *Cypripedium calceolus* (lady’s slipper orchid) in Europe, and we propose conservation strategies and directions for protective actions of its population on a regional scale. European *C. calceolus* populations have decreased significantly in the last two decades, in both number and size. A key result of the present study is an evaluation of the effectiveness of the Natura 2000 network across the European Union (EU) countries. Northern and/or mountainous countries present higher percentages of potentially suitable areas within the Natura 2000 network. Finland and the United Kingdom are the exceptions to this rule. It is predicted that, due to global warming, the coverage of niches suitable for *C. calceolus* will decrease in countries in which now-healthy colonies exist. However, as plant species can occur in micro-sites with suitable environmental conditions (e.g., microclimate, vegetation, soil factors) which cannot be predicted as suitable at coarser spatial resolutions, conservation efforts should be focused on management of local healthy populations. For the effective protection of *C. calceolus* in Natura 2000 sites, the participation of experts in botany, including orchid biology, is necessary at several stages.

## 1. Introduction

Since the end of the 18th century, it has been realized that anthropogenic effects on the natural environment are often destructive, and that action should be taken to conserve animal and plant species. Nowadays, the European Union (EU) Natura 2000, a network of protected areas designated under the Birds and Habitats Directives (2009/147/EC and 92/43/EEC, respectively), has been established across all 27 EU countries (the UK is considered a member state for the purposes of this paper). With more than 25,000 sites, covering over 18% of the EU’s land area and more than 8% of its marine territory, Natura 2000 is the largest coordinated network of protected areas in the world. More than 1000 animal and plant species, as well as 200 habitat types, as listed in the Directives’ Annexes, should be protected and appropriately managed to conserve EU biodiversity [1].

Of the orchids that have been recorded in EU countries, four species are listed in Annex II of the Habitats Directive: *Cephalanthera cucullata* Boiss and Heldr., *Cypripedium calceolus* L., *Liparis loeselii *(L.) Rich., and *Ophrys lunulata* Parl. Of these, *C. cucullata* and *O. lunulata* are endemics of Crete and of Sicily (and some nearby islands), respectively [2]. By contrast, *L. loeselii* is widely distributed in the northern hemisphere but is found only in fens, bogs, and dune slacks; its habitat specialization makes it extremely rare and threatened [3]. *Cypripedium calceolus* is a Eurasian taxon which also occurs from the Crimea through Siberia to Mongolia, China, and Japan [4,5]. It is found in large parts of northern Asia as far east as the Primorye Region of Russia [6], and it has recently been found in a totally isolated area in Algeria [7]. In Europe, the center of this species’ occurrence is in central and northern parts of the continent, while it is totally absent in the evergreen Mediterranean zone [8].

Across its global range, *C. calceolus* is regarded as a species of least concern according to the International Union for Conservation of Nature (IUCN) [9]. Notwithstanding its global status, in some countries it has been characterized as a species fulfilling the criteria for inclusion in threatened categories (for details see [10]). Moreover, *C. calceolus* is included under Appendix I of the Convention on International Trade in Endangered Species of Wild Fauna and Flora (CITES), under Appendix I of the Convention on the Conservation of European Wildlife and Natural Habitats (Bern Convention), is fully protected under Schedule 8 of the Wildlife and Countryside Act 1984, and is listed on Schedule 4 of the Conservation Regulations [3,11]. Information about its status in European countries can be found in Kull et al. [10], but in other territories it is often very rare and threatened with extinction, as in the Crimea, where it grows only in a single locality [12]. 

As a species with a high conservation value, *C. calceolus* is the only European orchid species for which an Action Conservation Plan has been published [8]. Despite its high protection status, *C. calceolus* is one of the European wild orchid species that has significantly declined during last two decades [8,9]. This is largely due to external factors causing disturbances of habitat conditions [13]. Progressive habitat loss, ending of traditional forest management (e.g., coppicing, small clearings), illegal digging-up for gardens, and improperly carried-out in situ conservation measures have reduced both the number of populations and the population sizes in Poland [14,15], as well as in other European countries [8]. 

Although, the aforementioned threats are expected to affect *C. calceolus* directly, through the reduction of its populations or of the number of individuals in those populations, the effects of the climate change are also expected to affect native plant species. Specifically, the recently recorded increase in summer temperatures, drought, large-scale fires, and lack of snow in winter tend to cause declines in the distribution ranges of several plant species, or to force them to migrate towards northern latitudes and higher altitudes [16,17]. Kolanowska and Jakubska-Busse [15], in their study of the effects of climate change on *C. calceolus* found that its range will decrease by ca. 30–63% by 2070. This is a big challenge for biodiversity conservation, especially when protected areas are in the lowlands and their environmental heterogeneity is low [18]. Regarding the effectiveness of the Natura 2000 network under future climatic conditions, Araújo et al. [19] found that areas of suitable climate will decrease for a significant number of the species of European concern occurring in Natura 2000 areas. In the light of future climate change, the effectiveness of the Natura 2000 network in conserving species of European concern could also be enhanced by a set of management measures that would ensure the existence of a variety of micro-habitats, suitable for most of these species.

Plant species are expected to respond to such changing environments in different ways. Thus, detailed knowledge of a species’ ecology [20], its current threat and conservation status, and the appropriate management requirements are essential for effective conservation in the future which will have to happen through a quick response from scientists, conservationists, forest managers and all other stakeholders. 

The aims of the present study are: (1) to summarize the conservation status, major threats, and threatening processes to the natural populations of *C. calceolus* in different geographical regions; (2) to explore the effectiveness of the Natura 2000 network of protected areas under current and future climatic conditions; (3) to identify the characteristics of the habitats and plant communities in which *C. calceolus* grows in the center of its distribution; and (4) to provide examples of good management practice across its European distribution range. 

We believe that our conclusions can improve *C. calceolus* conservation in Europe significantly, and that successful conservation of such an iconic species can inspire and support conservation for European biodiversity overall.

## 2. Materials and Methods

### 2.1. Study Species

*Cypripedium calceolus* L. 1753, the lady’s slipper orchid, is a long-lived, rhizomatous, terrestrial, cross-fertilized plant species e.g., [21,22,23], which grows in lightly shaded deciduous and mixed woodland (rarely in full sunlight, often in coppicing forests) and in meadows, predominantly on calcareous soils [23], as well as in deciduous and coniferous forests with an understory of grasses and other herbs, in forest clearings and lean pastures, often near stream banks, and in bushy hillsides. At the southern limits of its distribution it is found mostly in beech forests at montane and submontane elevations [8,24] and Appendix A. 

Its altitudinal preferences vary from country to country, correlated with the latitudinal gradient. Specifically, its altitudinal range starts from the mid-elevation zone (e.g., Serbia, 1350–1650 m a.s.l.; Croatia, 500–1700 m a.s.l.); it occurs at higher altitudes in southern countries (e.g., Bulgaria: 1340 m a.s.l.), while towards the northern European countries it can be found from the lowlands up to 2200 m a.s.l. [25]; e.g., Finland, 10–262 m a.s.l.; Denmark, 75 m a.s.l.; Britain, 150–260 m a.s.l.; Poland, 77–1046 m a.s.l.; Germany, 10–1540 m a.s.l.). This differentiation indicates that conservation practices and measures should be organized according to location. 

### 2.2. Conservation Status and Threats that Cypripedium calceolus Faces in Europe

To identify the major threats for *C. calceolus* in Europe, and to propose appropriate management measures, we used information obtained from a variety of sources published in the years 1993–2020—e.g., books, webpages, research articles—with expert knowledge used in cases where the necessary information was not otherwise directly available. The information that was used included (a) the most up-to-date IUCN threat category of *C. calceolus* for all European countries e.g., [26], Appendix B, (b) the criteria of the IUCN that *C. calceolus* meets in each country: A—population reduction, B—geographic range, C—small population size and decline, D—very small or restricted population, E—quantitative analysis [27], and (c) the factors that threaten the existence and survival of *C. calceolus*, as: collecting, clear cutting of the woods, grazing, damage by rodents, changes in the dominant tree species, habitat destruction, trampling, road construction, global heating, habitats drainage, forest and grassland fires, eutrophication, animal pests, grassland mowing or other stochastic events. However, data concerning the criteria of the IUCN and/or specific threats for *C. calceolus* were not available for all countries and some of the criteria could not be applied without presence of historical data. In the case of Ukraine, where IUCN criteria were not available, we tried to determine these on the basis of available chorological and population data [28,29]. To identify clusters of countries with a high degree of similarity in the threats that *C. calceolus* faces, we applied an unweighted pair group method with arithmetic mean (UPGMA) cluster algorithm using the Bray–Curtis similarity index. The UPGMA method was performed in R v. 4.0.2, using the vegan package [30].

### 2.3. Effectiveness of the Natura 2000 Network in Conserving Cypripedium calceolus 

The degree of effectiveness of the Natura 2000 network was explored using the published results of Kolanowska and Jakubska-Busse [15] regarding the current and future potential distribution of *Cypripedium calceolus*. Specifically, in their study Kolanowska and Jakubska-Busse [15] used maximum entropy techniques [31,32,33] to predict the potential distribution of this orchid. They used 12 bioclimatic variables (bio1: annual mean temperature; bio2: mean diurnal range; bio3: isothermality; bio4: temperature seasonality; bio5: max temperature of warmest month; bio8: mean temperature of wettest quarter; bio12: annual precipitation; bio13: precipitation of wettest month; bio14: precipitation of driest month; bio15: precipitation seasonality; bio18: precipitation of warmest quarter; bio19: precipitation of coldest quarter) at a spatial resolution of 2.5 min (approximately 22 km), and their predictions were based on the Community Climate System Model (CCSM4) for the year 2070, using four available representative concentration pathways (RCPs: rcp2.6, rcp4.5, rcp6.0 and rcp8.5). To explore the importance of the Natura 2000 network of protected areas, we used the sites of community importance (SCIs), as well as the special areas for conservation (SACs) downloaded from the European Environment Agency. The list of Natura 2000 habitats in exemplary European countries was prepared on the basis of published data e.g., [12,34,35,36,37,38,39]; for details see Appendix A.

Due to their coarse spatial scale, direct use of the predicted potential distributions of *Cypripedium calceolus* [15] would not allow an accurate estimation of the effectiveness of the Natura 2000 network in conserving *C. calceolus* under current and future climatic conditions. To overcome this problem, we increased the spatial resolution by resampling the initial raster layers of the predictions to 30 s resolution (approximately 1 km^2^). As the output of the species-distribution models is a continuous habitat-suitability map, we had to convert the habitat-suitability maps into binary maps using a cut-off value as a threshold in order to identify areas that are potentially suitable for *C. calceolus*. In accordance with the results presented by Kolanowska and Jakubska-Busse [15], the habitat-suitability cut-off value was set as 0.4, with values at or above that threshold set as presences and values below as absences. 

Additionally, the vector file corresponding to sites of community importance and to special areas for conservation was converted into a raster layer with the same spatial resolution and extent as the resampled raster layers of the binary predictions. We then converted this modified raster layer into a point shapefile, wherein points correspond to the centroids of the resampled raster grids (raster layers of the predictions). As final steps, we extracted values of the predictions of *C. calceolus* under current and future climatic conditions for each point of the point shapefile and measured the number of grid cells of the Natura 2000 network for each of the EU countries corresponding to the presence of *C. calceolus*.

## 3. Results

The IUCN threat status of *Cypripedium calceolus*, as well as the criteria that it fulfills, are presented in Table 1 for each European country. In total, the species is present, or has been recorded, in 35 countries or areas, including the European part of Russia and in Crimea, whereas it has never been recorded in seven countries, where it is, therefore, considered absent (Iceland, Ireland, Cyprus, Portugal, North Macedonia, and Malta). It is extinct in five countries (Table 1), and classified as threatened (Critically Endangered (CR), Endangered (EN), or Vulnerable (VU)) in 22 out of the 35 countries of Europe (i.e., 62%).

The most common threat category was that of ‘Vulnerable’ (11/35 countries; 31%), followed by ‘Critically Endangered’ (6/35; 17%) and ‘Endangered’ (5/25; 14%). Moreover, in another five countries, *C. calceolus* was classified in the ‘Near Threatened’ category, whereas it is considered as of ‘Least Concern’ in only three European countries (9%; Table 1). 

The classification of a species in any of the three threat categories of the IUCN (CR, EN, and VU) is based on a series of five different criteria (A, B, C, D, and E). As can be seen in Table 1, the most common criteria are A and B (*C. calceolus* meets these criteria in 41% of the countries where it was classified as threatened), followed by criteria D and C (27%, 23% of those countries). Criterion E was not met in any of the countries where the species has been classified in a threat category.

**Figure 1:**
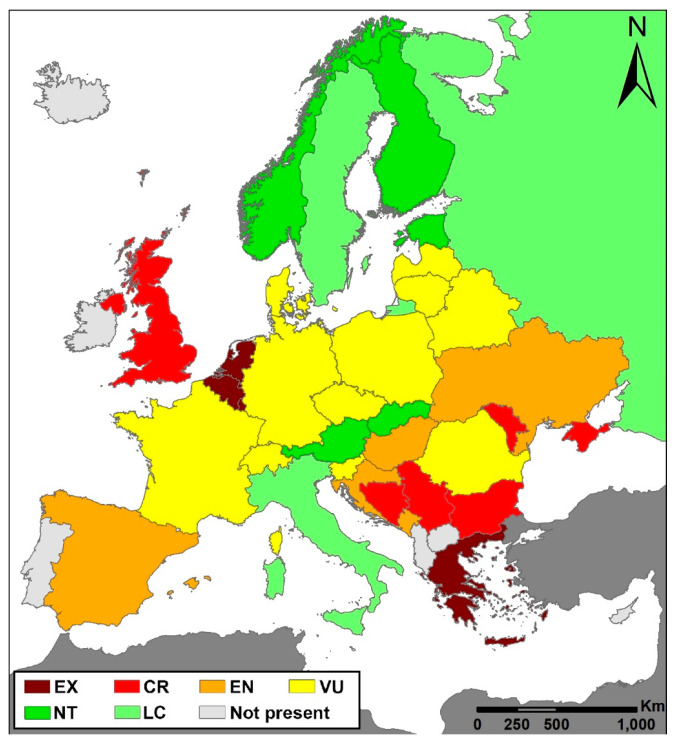
Map of Europe presenting the IUCN threat category of *Cypripedium calceolus* in each country. The map was created using the projected coordinate system “Lambert_Azimuthal_Equal_Area”.

With the exception of the United Kingdom, where *C. calceolus* is characterized as critically endangered, its IUCN threat status is higher towards the southern European countries (Figure 1). In most countries of the Balkan Peninsula, *C. calceolus* is classified in the highest IUCN threat categories (i.e., critically endangered or vulnerable); it is absent from the southernmost countries, and extinct in Greece. By contrast, as a species of northern origin *C. caleolus* is of least concern or near threatened towards the northern-European countries, and especially in Scandinavia. A second, but smaller, core of countries where the species has been characterized as near threatened or of least concern is in central Europe, specifically Italy, Austria, and Slovakia (Figure 1).

Where possible (data from 30 countries were available), the factors offering the greatest threats to *C. calceolus* were identified (Table 2). Collecting (reported as a threat in 25 countries), vegetation succession (20 countries), and clear cutting of the woods (19 countries) are the most common threats, followed by grazing and damage by rodents (7 countries), changes in the dominant tree species (6 countries), and habitat destruction (6 countries). On the other hand, eutrophication, animal pests (insects, snails), inappropriate grassland mowing, fragmentation, and other stochastic events are the least common factors regarding the number of countries in which these have been identified as threats. 

**Figure 2:**
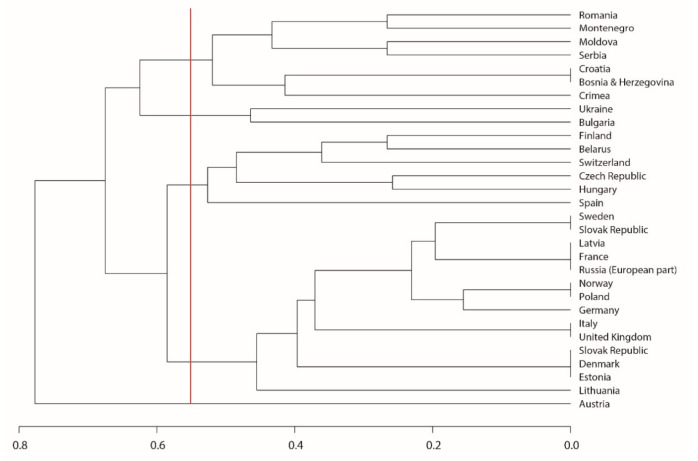
Results of hierarchical cluster analysis (unweighted pair group method with arithmetic mean, UPGMA) showing the degree of similarity of European countries where *Cypripedium calceolus* is present, using Bray-Curtis distances of the factors that have been identified as threats at each country. The vertical red line denotes the limit that led to the identification of the five groups of countries.

Five groups of countries can be distinguished in which the factors that threaten *C. calceolus* are identical or similar (Figure 2). Austria stands alone in having eutrophication and changes in the dominant tree species identified as the major threats for *C. calceolus*; these factors were minor elsewhere, and reported from only a few other countries.

**Figure 3:**
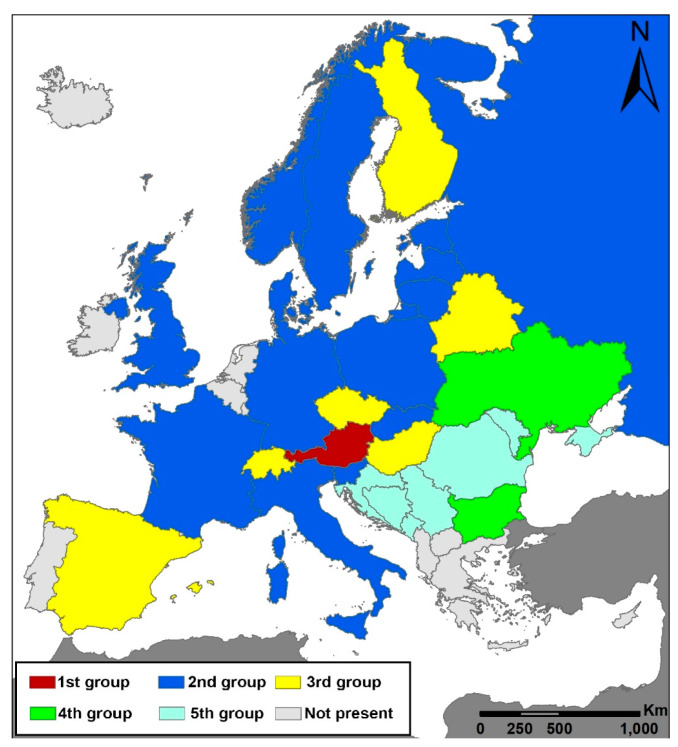
Groups of countries in which *Cypripedium calceolus* faces similar threats. The groups of countries have been identified on the basis of an unweighted pair-group method with arithmetic mean (UPGMA) cluster algorithm. The map was created using the projected coordinate system “Lambert_Azimuthal_Equal_Area”.

Regarding the spatial distribution of the threats, towards the south of the orchid’s distribution range (in the Balkans) threats are mostly related to collection, and secondarily to the clear cutting of the woods where *C. calceolus* occurs (Table 2, Figure 3). Bulgaria and Ukraine were grouped together because, aside from collecting, forest fires constitute a serious threat for *C. calceolus*. In the central and northern parts of the range, collecting, clear cutting of woods, and vegetation succession have been identified as major threats. However, within this single large group, two smaller groups can be recognized (2nd and 3rd groups). While collecting, clear cutting, and vegetation succession are the most serious factors in more than half of these countries, in the remainder (Hungary, Spain, Finland, Belarus, Czech Republic, and Switzerland) other aspects, such as trampling and grazing damage by rodents, are also important. It is essential to understand the grazing herd size in order to establish a grazing plan with no overgrazing [40].

Despite an awareness that the occurrence of *Cypripedium* *calceolus* is limited by many environmental attributes (e.g., habitat, geology, and biotic interactions), as well as by historical factors, we elected to calculate the potentially suitable area for *C. calceolus* within the Natura 2000 network of EU members, constrained only by current and future climatic conditions (Table 3). The results necessarily overestimate the actual candidate areas, because ecological conditions in many Natura 2000 sites do not meet *C. calceolus* environmental requirements; nevertheless, this calculation helps better inform an understanding of the importance of changing climatic conditions. Under current conditions, the Czech Republic, Austria, Luxembourg, and the Slovak Republic have the greatest percentages of potentially suitable areas within their Natura 2000 networks, while Greece, Spain, Finland, the United Kingdom, and Belgium are characterized by the lowest percentages. However, under future climatic conditions, as based on the Community Climate System Model for the year 2070 (CCSM4; RCPs rcp2.6, rcp4.5, rcp6.0 and rcp8.5), the suitability of the Natura 2000 network will be significantly reduced in most countries. Under the worst representative concentration pathways (rcp8.5), Austria, Sweden, and the Slovak Republic will have the highest percentages of potentially suitable habitats within their networks of protected areas, whereas suitable areas for *C. calceolus* disappear in many central and northern European countries (e.g., Estonia, Latvia, Lithuania, Hungary).

In Natura 2000 sites, populations of *C. calceolus* are usually restricted to specific areas. Table 4 lists the habitats that can ensure the survival of this species within the Natura 2000 network. Some populations of *C. calceolus* also occur in non-native spruce monocultures—commercial forest cultures which have replaced the original natural habitats. These populations are usually unprotected and are under exceptionally high threat by commercial forestry. 

This section may be divided by subheadings. It should provide a concise and precise description of the experimental results, their interpretation, as well as the experimental conclusions that can be drawn.

## 4. Discussion

### 4.1. Conservation Status

Despite *Cypripedium calceolus* being listed as a species of least concern in the IUCN Red List, with population trends described as stable at the global scale [9], we found that this species is classified as threatened (critically endangered, endangered, or vulnerable) in most European countries, where both the numbers and sizes of populations have decreased significantly over the last two decades. This could be attributed to the intensive environmental stress, habitat losses and fragmentation, small population sizes, etc., which caused a great reduction in its range. In Europe, *C. calceolus* is widespread but has undergone severe declines in the past, especially due to its collection by enthusiasts [9]. For example, according to Bilz [41], in the Czech Republic there are many localities but they are generally very small in size and the populations are fragmented, forming small scattered patches [35], whereas in Hungary, it occurs at no more than eight localities where it was formerly known from more than 20. This decrease has happened during the last century. The number of localities has remained constant over the last 10 years but the number of individuals has been continuously decreasing. Moreover, in Poland, there are historical records for over 200 localities in the lowlands, but most of them have been lost especially in western Poland. Similarly, in Norway, a population decline of 15–30% has been observed in the past and is assumed to continue in the future [1]. Generally, the populations are declining in parts of its range but are stable or increasing in other parts due to conservation measures that were taken [20]. The aforementioned arguments clearly demonstrate that conservation measures for the protection of *C. calceolus* at a pan-European level should be focused on its threat status at a country level. In this sense, the last IUCN global assessment of this species, which was conducted in 2014, cannot provide information of crucial importance regarding its regional status and threats. Moreover, as the specific assessment was based predominantly on data from Europe [9], we can suppose that lack of current data, as well as long-term monitoring data describing the situation in detail outside of Europe, are not available. This could also partly explain the imbalance between the conservation status of *C. calceolus* in Europe and worldwide. There is a high probability that there would be significant differences between its current status in different regions globally (e.g., Europe vs. Asia), as well as among the different European countries (e.g., southern vs. northern).

Differences can be discerned even between regions in a single country. In some cases, we found that the level of threat on a national scale may not correspond to the level in particular regions. For example, the species is considered nationally vulnerable in Poland, but has a higher threat level in seven out of 11 provinces e.g., [42,43]. It is identified as vulnerable and near threatened in two regions, and is absent in two other regional red lists. In several central and northern European countries, although the climatic conditions are suitable for *C. calceolus* (as well as for several other orchid species), the species has declined because of changes in land cover (e.g., deforestation, agricultural intensification) and in forest-management practices (e.g., changes in the dominant tree species, light conditions, density of shrubs) [44,45]. By contrast, in southern-European countries (e.g., Italy, Spain), *C. calceolus* is restricted to the northern parts of these countries, or to mountainous areas where the climatic conditions are suitable. 

### 4.2. Threats

European countries differ not only in current *Cypripedium calceolus* conservation statuses but also in the major threats to this species [8] (Table 2). In Natura 2000 sites, populations of *C. calceolus* are usually restricted to specific areas. Table 4 lists the habitats that can ensure the survival of the species across the European Natura 2000 sites. It is worth noting that *C. calceolus* also occurs in other plant communities (see Appendix A) that were not intended for protection due to inconsistency with the description of the habitat in the Council Directive 92/43/EEC of 21 May 1992 on the protection of natural habitats and wild fauna and flora (Council Directive 92/43/EEC). In general, threats to the populations of *C. calceolus* can be divided into two basic groups: natural and anthropogenic. 

Natural factors include, for example, spring frosts, drought, or vegetation succession. Early-spring ground-level frosts can cause permanent, irreversible damage to *C. calceolus* inflorescences or to single flowers, and this problem is very harmful to populations growing in open meadows (compare those occurring within forests, where the minimum temperatures are always higher). This can be particularly significant towards the latitudinal or elevational limits of the species [46]. Drought events can also be harmful; Corkhill [47] noted that *C. calceolus* is sensitive to drought, and that young seedlings, especially, require constant moderate moisture. Although the aforementioned threats relate to the climatic conditions of sites where *C. calceolus* occurs, vegetation succession was classified as being among the most serious threats for this species in Europe. The disappearance of *C. calceolus* populations may be the result of the gradual overgrowth of trees, shrubs and herbaceous plants that occur at the sites. Based on our observations, *C. calceolus* populations in SW Poland are threatened by *Crataegus* L. spp., *Rubus fruticosus* L., *Aegopodium podagraria* L., and *Fraxinus excelsior* L. Such changes in the vegetation cover affect light conditions, which in turn significantly affect the viability of *C. calceolus* populations. Previous research has shown that reduction of light intensity caused by forest overgrowth can lead to an extension of the dormant period and postponement of the flowering period of this species [48]. 

Anthropogenic factors are usually stronger and may have fatal consequences. According to the IUCN, major threats to this species are habitat destruction, agriculture intensification, ecosystem modification, and inappropriate forest management (such as clear cutting, logging, and wood harvesting, use of herbicides and pesticides, equipment use that can severely compact the soil, agricultural and forestry effluents, road and trail construction), as well as collecting from the wild [9]. Collecting, reported as a major threat in as many as 25 European countries, is inexcusable in the 21st century, but digging and replanting into gardens unfortunately still occurs. In this way, unique meadow populations are destroyed—for example, the population in the ‘Babylon’ meadow in the eastern Sudetes (SW Poland) was irretrievably destroyed in 2016. It is hard to believe that specimens of *C. calceolus,* an iconic species protected by both national and international laws, are still being collected for private gardens and for herbaria. Burning of meadows and grasslands may also be one of the key factors in destroying the structure of the population and in limiting the range of the species at a regional scale. Other factors affecting the species habitats should also be taken into account, such as digging (meant here as intentional destruction, not related to collecting) and landslides caused by erosion as a result of human activity or natural disasters like drought, floods and hurricanes, which were described as stochastic events (Table 2).

Specific forest-management practices can severely threaten *C. calceolus* [41]. Among such practices, clear-cutting of woods threatens *C. calceolus* populations in many countries. Large-scale deforestation, especially when the dominant and native tree species are replaced, is a controversial practice that is widely applied in commercial forestry in many European countries. One of the largest *C. calceolus* populations in the SW Poland was destroyed as a result of clear-cutting in 2017, on state property, and compensation was considered unnecessary. On the other hand, clear cutting in the species’ localities is prohibited in some countries (e.g., Slovenia, Italy), and others will follow soon as the result of a new EU Forest Strategy [49]. Following national and EU legislation, local regulations for forest management should be applied in protected areas and in Natura 2000 sites in all EU countries. In particular, data from official *C. calceolus* monitoring should be updated and made available as open-access.

### 4.3. Conservation Strategy

Only after the commencement of detailed studies on *Cypripedium calceolus* does the regional conservation status of its populations have a chance of being improved. It is necessary to protect habitats and micro-sites where this species occurs, and to map its actual distribution, in particular by searching old-growth forests with a predominance of beech, but also in oak--hornbeam forests and other plant communities suitable for the species (Table 4, Appendix A). Since *C. calceolus* has a wide ecological latitude and is not confined only to one specific plant community, it is worth establishing co-operation with foresters who usually have knowledge of the locations of the most valuable plant species. Historical data should also be analyzed, as there are instances of the rediscovery of numerous *C. calceolus* populations, even after as long as 80 years [34]. Ultimately, it is necessary to check regularly all known populations of the species, including those that survive in conifer plantations and on private land. Accordingly, a list of both recommended and inappropriate conservation measures is presented in Table 5, based on data collected concerning practices that focus on the protection of the species.

A key finding of the present study is the degree of effectiveness of the Natura 2000 network across the EU countries. Northern and/or mountainous countries present the highest percentages of potentially suitable areas within the Natura 2000 network. Finland and the United Kingdom constitute two exceptions to this rule. The lower habitat suitability of the networks of these two nations could be attributed to the sampling bias of the species records used to build their models [15]. This might be also be the case for other countries where the species is absent (e.g., Belgium, the Netherlands). 

The fact that, under future climatic conditions, *C. calceolus* is expected to decline in several countries in which healthy colonies currently exist clearly demonstrates that appropriate conservation actions should be applied for its survival. Such actions should include appropriate management of the sites where *C. calceolus* is present (e.g., restrictions on clear-cutting, control of vegetation succession; [45], improvement of site conditions where the species was recorded in the past, and establishment of seedlings or young plants [50]. 

However, it might be hypothesized that the predictions of Kolanowska and Jakubska-Busse [15] are only indicative of the actual distribution of *C. calceolus*. This could be attributed to the environmental predictors used by Kolanowska and Jakubska-Busse [15], as well as to the spatial resolution of the analyses. Although Kolanowska and Jakubska-Busse [15] exclusively used bioclimatic variables to build their models, for such a wide distribution area (i.e., Europe) this is not a restriction. This is because species distributions at broad geographical scales are mainly driven by climatic factors [51], while the inclusion of land-cover variables in bioclimatic models does not improve their predictive accuracy, according to Thuiller et al. [52]. By contrast, it is known that plant species can occur in micro-sites with suitable environmental conditions (e.g., microclimate, vegetation, soil factors) that cannot be predicted as suitable using a coarser spatial resolution [53]. 

It is well known that the results of species-distribution models can be used to guide field surveys in order to find populations of known or rare species and to set conservation priorities (for more information see [54]). Field surveys towards areas of higher habitat suitability are very important in recording new populations of rare species. This is especially true for orchids, whose distributions are affected not only by abiotic factors, but also, for several species, by the distribution of other organisms (pollinators, mycorrhizal fungi; [16,55]). Moreover, conservation actions might be focused on those areas where greater possibilities for the future existence of selected species are observed.

**Figure 4:**
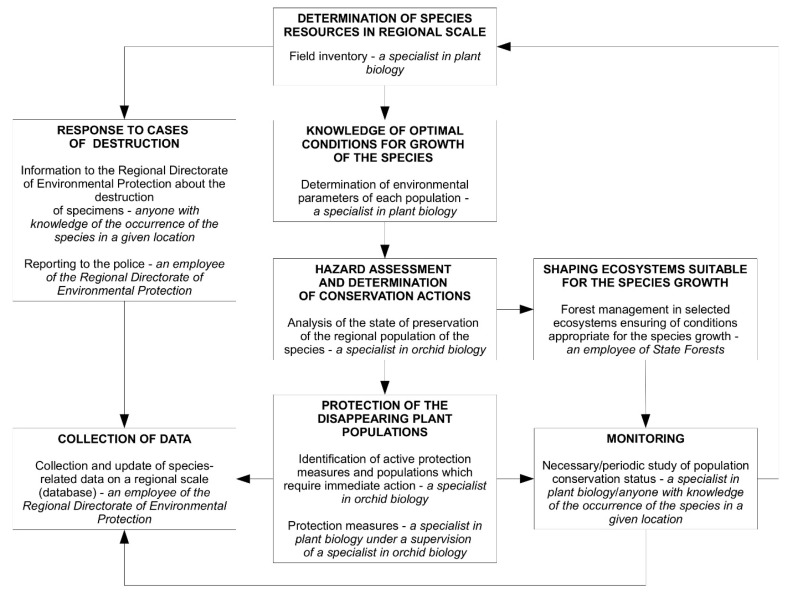
Proposed protective-action directions of *Cypripedium calceolus* populations on a regional scale.

Observations of *C. calceolus* from various countries indicate its limited occurrence, and the need to develop effective methods of protection [56]. The species maintains populations under certain habitat conditions, but different population sizes and plant communities each require a special approach and uses of different conservation measures; each population should, therefore, be treated individually. The human influence on species is generally negative, manifested by actions that have led to the decline of populations in many European countries. The actions undertaken are usually limited to passive protection, until the destruction of individuals due to natural or anthropogenic factors. For the effective protection of *C. calceolus* in Natura 2000 sites, the participation of experts in botany, including orchid biology, is necessary at several stages. Their participation is a requirement for the assessment of resources, habitat conditions, threats, and protective actions in each Natura 2000 site where *C. calceolus* is found. The results should lead to further activities at the regional scale, including the shaping of ecosystems (usually aimed at improving the condition of the Natura 2000 habitats) and implementation of necessary legal actions. Populations of the species are difficult to protect due to its complex biology and, while listed in Annex II of Council Directive 92/43/EEC, it requires special attention in terms of forestry. Since the conservation potential of *C. calceolus* varies by country, there is no comprehensive management system for this species. Based on experience gained, directions for the protection of *C. calceolus* leading to the best protection of its population were developed, presented in Figure 4.

## 5. Conclusions

The key role of scientists is in the identification of plant communities and of optimal environmental conditions in which *Cypripedium calceolus* grows. This should be a guideline for those entities responsible for the management of areas where this species is found. It is necessary to develop methods and to implement a pattern of handling the occurrence of *C. calceolus* because, in many European countries, this is still a minor issue compared to, e.g., the amount of wood that must be obtained from the forest in which the species grows. Projects for regional conservation of this species, developed by scientists with financial support from the region’s authorities (involving all stakeholders—e.g., forest owners, managing companies, local communities), can save many *C. calceolus* populations and enhance their viability, starting from a detailed inventory, risk assessment, development and implementation of protective measures, and further observations (Figure 4). In any case, comprehensive efforts must be made to maintain the ecosystems in which lady’s slipper grows or could grow. Actions undertaken by foresters should not be irreversible but cyclical, in close co-operation with scientists. Monitoring activities [57] will allow to a better understanding of the threats affecting the species and population dynamics. This information will be of great help for the possible implementation in the future of in situ and/or ex situ conservation actions [57,58] allowing for effective species protection at the regional level.

Updating the global occurrence, current conservation status, and demography of *Cypripedium calceolus* populations will also play an important role in the protection of this orchid species. Failure to update the chorological data, or the introduction of imprecise data, distorts our knowledge of the actual state of its population, and may make it difficult to conduct effective protective measures.

## Figures and Tables

**Table 1:** Threat status in European countries, by International Union for Conservation of Nature (IUCN) categories.

Country/Area	IUCN Category	IUCN Criteria				
		A	B	C	D	E
Belgium, Greece, Liechtenstein, Luxemburg, The Netherlands	EX	-	-	-	-	-
Bulgaria, Crimea	CR	-	1	1	1	-
Serbia	CR	-	1	1	-	-
Moldova	CR	-	1	-	1	-
Bosnia and Herzegovina	CR	1	-	-	-	-
United Kingdom	CR	-	-	-	1	-
Croatia, Hungary	EN	1	-	-	-	-
Montenegro	EN	-	-	-	1	-
Ukraine	EN	-	1	-	-	-
Spain	EN	-	-	1	-	-
Germany	VU	1	1	-	-	-
Belarus, France, Latvia, Lithuania, Slovenia	VU	1	-	-	-	-
Poland, Romania, Switzerland	VU	-	1	-	-	-
Czech Republic	VU	-	-	1	-	-
Denmark	VU	-	-	-	1	-
Austria, Estonia, Finland, Slovak Republic, Norway	NT	-	-	-	-	-
Italy, Russia (European part), Sweden	LC	-	-	-	-	-
EX: Extinct; CR: Critically Endangered; EN: Endangered; VU: Vulnerable; NT: Near Threatened; LC: Least Concern						
Countries where *C. calceolus* is absent: Albania, Cyprus, Iceland, Ireland, Malta, North Macedonia, Portugal						

**Table 2:** Factors that have been identified as threats for *Cypripedium calceolus,* by country.

	Collecting	Vegetation Succession	Clear Cutting of the Woods	Grazing, Damage by Rodents	Changes in the Dominant Treespecies (e.g., Reforestation with Pines or Spruce)	Habitat Destruction	Trampling	Road Construction	Global Heating	Habitats Drainage	Forest and Grassland Fires	Eutrophication	Animal Pests (Insects, Smails)	Grassland Mowing	Other Stochastic Events
Austria		●			●							●			
Belarus	●	●	●	●	●	●	●	●		●					
Bosnia and Herzegovina	●														
Bulgaria	●						●		●		●				
Crimea	●							●							
Croatia	●														
Czech Republic	●	●	●	●			●							●	
Denmark		●	●												
Estonia		●	●												
Finland	●	●		●		●		●		●					
France	●	●	●												
Germany	●	●	●		●	●									
Hungary	●	●	●	●			●		●				●		
Italy	●	●													
Latvia	●	●	●												
Lithuania		●	●	●	●										
Moldova	●		●						●						
Montenegro	●		●												
Norway	●	●	●		●										
Poland	●	●	●		●						●				●
Romania	●		●	●											
Russia (European part)	●	●	●												
Serbia	●								●						
Slovak Republic	●	●	●			●									
Slovenia		●	●												
Spain	●			●			●	●							
Sweden	●	●	●			●									
Switzerland	●	●	●			●		●							●
Ukraine	●									●	●				
United Kingdom	●	●													
TOTAL	25	20	19	7	6	6	5	5	4	3	2	1	1	1	1

**Table 3:** Numbers of 1 × 1 km grid cells in the Natura 2000 network for each country, and percentage of potentially suitable areas for *Cypripedium calceolus* within the network. (RCP: representative concentration pathway).

Country	Grid Cells of the Natura 2000 Network	Potentially Suitable Area (% of the Natura 2000 Network)				
		Current Climate	Future Climatic Conditions (CCSM4)			
			RCP2.6	RCP4.5	RCP6.0	RCP8.5
Austria	8638	89.65	55.14	43.45	43.49	29.88
Belgium	2023	5.88	4.60	4.40	0.99	0
Bulgaria	48,054	15.57	3.03	1.93	1.47	0.02
Croatia	26,356	29.85	5.46	0.63	1.75	0
Cyprus	1217	0	0	0	0	0
Czech Republic	14,230	98.31	66.85	44.43	26.48	12.35
Denmark	5868	74.63	61.71	34.99	54.93	19.07
Estonia	9325	16.85	10.43	0.46	0	0
Finland	53,579	2.61	4.90	2.85	2.47	4.97
France	80,612	24.62	22.24	20.11	18.43	12.17
Germany	59,599	65.65	34.95	26.84	27.13	10.29
Greece	29,836	0.42	0.28	0.07	0.07	0
Hungary	21,865	8.82	0.03	0	0	0
Ireland	13,986	0	0	0	0	0
Italy	47,610	23.84	18.13	16.56	17.46	13.49
Latvia	1779	29.62	13.04	0.06	0	0
Lithuania	11,170	59.30	20.67	13.05	0	0
Luxembourg	738	89.02	57.32	70.05	58.67	12.87
Malta	43	0	0	0	0	0
The Netherlands	4813	9.27	26.01	15.62	11.68	0
Poland	58,730	57.01	12.92	5.50	6.73	2.66
Portugal	21,279	0	0	0	0	0
Romania	67,318	47.65	30.95	22.23	21.25	8.61
Slovak Republic	10,838	86.81	54.92	31.03	38.91	20.04
Slovenia	11,137	62.37	28.64	21.06	14.62	9.26
Spain	115,180	1.48	1.90	1.71	1.87	1.35
Sweden	83,859	23.07	33.56	26.14	24.70	22.93
United Kingdom	26,962	3.53	2.82	1.34	4.61	4.41

**Table 4:** List of Natura 2000 habitats with *Cypripedium calceolus* sites in exemplary European countries.

Habitat Type		Alpine and Subalpine Grasslands	Montane and Xerothermic Grasslands	Alternately Wet Meadows	Wet Screes	Beech Forests (Fertile, Calcified)	Oak-hornbeam Forests	Ravine Forests	Riparian Forests	Thermofilous Oak Forests
Natura 2000 Code		6170	6210 6520	6410	8160	9130 9150	9170	9180	91E0	91I0
Country	Crimea						●			
	Croatia					●				
	Czech Republic		●	●		●	●	●		●
	Germany		●			●	●			
	Poland	●	●			●	●			●
	Romania		●			●	●			
	Russian Federation (Europaean Regions)						●			
	Slovenia	●			●	●			●	

**Table 5:** Conservation strategy for *Cypripedium calceolus*.

Protective Actions for Natural Populations of *Cypripedium calceolus*
**Recommended**
Habitat protection Monitoring and population estimation Database with detailed information on the species habitat and population (including genetic structure) Exchange of knowledge between experts and standarization of protection procedures in all European countries Integration of the national areas hosting populations of *Cypripedium calceolus* in the European network for this species to exchange of information and cooperation for using the best techniques and strategies conservation, as well as in order to obtain funds for the elaboration conservation plan Education of local residents in the importance of the species protection Ex situ conservation An evaluation of administrative and statutory procedures
**Inappropriate**
Implementation of protective or economic activities without environmental supervision Determination of protective measures by unskilled persons Ignoring the possible occurrence of species in a poorly explored area Publishing of Global Positioning System (GPS) coordinates of *Cypripedium calceolus* population

## Data Availability

The data presented in this study are available on request from the corresponding author.

## Appendix A

**Habitats and communities of *Cypripedium calceolus* in different regions of Europe**

### Appendix A.1. Bulgaria

In Bulgaria, it inhabits mixed forests and *Pinus nigra* subsp. *pallasiana* forests. The forest phytocoenoses are formed by 120-year-old trees dominated by *Pinus nigra* and an admixture of *Abies alba* and *Fagus sylvatica*. In the shrub layer, undergrowth is created by the above species of trees, while underbrush consists of *Tilia platyphyllos*, *Acer hyrcanum*, *Fraxinus ornus*, *Sorbus torminalis*, *Cornus mas*, *Lonicera coerulea*, *Daphne mezereum*, *Rubus saxatilis* and *Clematis vitalba*. The grass layer is dominated by *Calamagrostis arundinacea*, with a significant share of *Pteridium aquilinum*, *Lilium martagon*, *Cephalanthera longifolia*, *Corallorhiza trifida*, *Polygonatum odoratum*, *Aremonia agrimonoides*, *Cardamine bulbifera*, *Clinopodium vulgare*, *Euphorbia amygdaloides*, *Fragaria vesca*, *Potentilla regis-borissii*, *Mycelis muralis*, *Helleborus odorus*, *Primula veris*, *Hieracium murorum*, *Pyrola chlorantha*, *Salvia glutinosa*, *Sanicula europaea*, *Viola odorata*, etc. There are also seedlings of *Fagus sylvatica*, *Acer hyrcanum* and patches of mosses [59].

### Appendix A.2. Crimea

In total, 15–20 localities in the Crimea were known, but all clustered rather locally in the Western part of the Crimean Mountains; currently the species is known from only one site on Boyka Mountain near Sokolinoye. Ecologically, it grows (or was growing) in Crimea in beech forests often mixed with hornbeam (*Carpinus*), usually near stream banks at 500–1100 m a.s.l. [12,60].

### Appendix A.3. Croatia

In this country *Cypripedium calceolus* was found on Velebit Mt, Bilogora Mt, Kalnik Mt, Žumberak Mt, in Gorski Kotar, Plitvice Lakes National Park, Krbava, and at Plješevica Mt, inhabiting most commonly *Fagus sylvatica* forests, especially the community *Fagetum croaticum australe* [24,61,62]. Recent studies on the Velebit mountain range indicated that *C. calceolus* grows at the edge of a beech forest together with the following species: *Astrantia major*, *Cardamine enneaphyllos*, *Dryopterix filix-mas*, *Maianthemum bifolium*, *Neottia nidus-avis*, *Paris quadrifolia*, *Polypodium vulgare*, *Pulmonaria officinalis*, *Sanicula europaea* and *Symphytum tuberosum*; in subalpine beech forests on sites of northern aspect together with *Astrantia major*, *Cerastium dinaricum*, *Dryas octopetala*, *Primula kitaibeliana* and *Aethionema saxatile*, as well as in mixed forests of *Fagus sylvatica* and *Abies alba*, with the following accompanied taxa: *Acer pseudoplatanus*, *Anthriscus nitidus*, *Astrantia major*, *Geranium phaeum* and *Picea abies* [62]. Furthermore, Nikolić and Topić [63] noted that *C. calceolus* occurs in Illyrian low-montane acidocline fir-beech forests (code 41.1C221) and Illyrian low-montane neutrophile fir-beech forests (code 41.1C222).

### Appendix A.4. Czech Republic

Recently there are about 80 locations of *Cypripedium calceolus* in the Czech Republic (www.biomonitoring.cz, accessed on 18 January 2021). They are located in lowlands, hills, foothills of termophytic, mesophytic and oreophytic regions of the country, i.e., all geographic regions except the Moravskoslezský, Plzeňský and Karlovarský region (for a map of distribution see www.pladias.cz, accessed on 18 January 2021).

This species usually grows in light forests and their edges, but some populations also survive in cultural spruce forests [64,65]. Oak-hornbeam forests, thermophiles oak forests, herb-rich beech forests and rarely also ravine forests are the most common habitats of this species [35]. However, it also occurs on non-forest habitats - in the humid variations of broad-leaved grasslands and in alternately wet *Molinion* meadows. *C. calceolus* is as a taxon mainly growing in forest borders and canopy gaps including forest roads, fallen trees areas, fires and clearings. It is usually found in forests with sparse herbaceous undergrowth.

### Appendix A.5. Germany

The species occurs in Germany from the Baltic Sea coast to higher altitudes in the Bavarian Limestone Alps. In Northeast Germany, the remaining populations are extremely threatened with extinction. The once individual occurrences on the Jasmund Peninsula on Rügen are now close to extinction. Here the species occurred in the chalk coast in orchid beech forests. Another remaining occurrence in Brandenburg consists of only a few sterile individual plants [66]. Size and stable occurrences only exist in central and southern Germany. Occurrences with several thousand individuals can still be found in Lower Saxony, Thuringia, Bavaria and Baden-Württemberg, but numerous occurrences only consist of small populations and individual plants. Kretzschmar [67] calculated an absolute decrease of 57% of the occurrence for Central Germany. In the meantime, however, the situation in many regions is likely to have deteriorated dramatically. As part of targeted protective measures (e.g., LIFE+ project “Diversity on limestone” in North Rhine-Westphalia [68], occurrences of *Cypripedium calceolus* were also stabilized and growth conditions improved again. In Germany, *C. calceolus* is a kind of semi-shady, grass and herb rich, often also moss-rich, more or less light deciduous and coniferous forest sites [38].

### Appendix A.6. Poland

*Cypripedium calceolus* is a plant species, which is generally not attached to a specific habitat or plant community. In the Polish lowlands, it prefers forest habitats of varying degrees of preservation, but it also occurs in the forest edge communities, like thickets and roadsides. In highlands and mountainous conditions, it is known from grasslands, usually partially shaded with trees or bushes. *C. calceolus* habitats are characterized by good light availability and a high content of calcium carbonate in the substrate. The species most often grows on rendzinas consisting of dolomites, limestones and marls, rarely on brown soils, black molds and alluvials, in lowlands it was also observed on boulder clays. In upland areas, the species grows on moist soils, characterized by an undertone of water rich in calcium carbonate, on gentle slopes it occurs on shallow soils, with present stony fraction and even rock rubble. In mountainous regions, it inhabits steep slopes on shallow humus rendzinas, formed on heavy clays. If it grows in forest communities, the plants are characterized by a relatively lax inflorescence. It is observed not only in 120- to 150-year-old forests, but also in 40- to 50-year-old woodland, and in forest gaps created due to cutting. Less often it is found in open areas, usually in the vicinity of open undergrowth of trees or shrubs [34].

In climatic and geographical conditions in Poland, *C. calceolus* occurs mostly in forest areas characterized by beech and oak stands, with an admixture of hornbeam, ash, linden, maples, as well as spruce, larch, pine and sometimes yew [34]. It is also found in birch, pine and spruce plantations on beech or fertile oak-hornbeam habitats [34]. It is usually observed at the edges of forests, where it grows alongside roads. In the undergrowth of fertile oak forests, it is most often accompanied by: *Anemone nemorosa*, *Asarum europaeum*, *Daphne mezereum*, *Epipactis helleborine*, *Galeobdolon luteum*, *Galium odoratum*, *Hedera helix*, *Melica nutans*, *Viola reichenbachiana* and *Viola mirabilis*, while in fertile beech forests, it grows in vegetation patches with: *Aquilegia vulgaris*, *Carex digitata*, *Carex montana*, *Hypericum montanum*, *Lathyrus niger*, *Lilium martagon*, *Polygonatum odoratum*, *Pyrola rotundifolia*, *Pyrola secunda*, *Sanicula europaea* and *Vincetoxicum hirundinaria* [69,70]. In “orchidaceous” beech forests, it is also accompanied by: *Cephalanthera damasonium*, *Cephalanthera rubra*, *Corallorhiza trifida*, *Epipactis helleborine* and *Neottia nidus-avis*. To date, several forest associations with confirmed occurrence of *Cypripedium calceolus* have been identified in Poland, which are: *Potentillo albae-Quercetum* (*Quercetalia pubescenti-petraeae* order), *Stellario-Carpinetum*, *Galio-Carpinetum*, *Tilio-Carpinetum* (*Carpinion betuli* alliance), *Carici albae-Fagetum*, *Dentario enneaphyllidis-Fagetum*, *Fagus sylvatica-Hypericum maculatum* (also described as *Taxo-Fagetum*) and *Fagus sylvatica-Cypripedium calceolus* (*Fagion sylvaticae* alliance), the last of which was identified as a unique humid calcareous beech forest, known from only one site in the country. In terms of beech forests, associations with *C. calceolus* were not always identified, but were generalised at level of sub-alliances: *Galio odorati-Fagenion*, *Cephalanthero-Fagenion* or *Dentario glandulosae-Fagenion* (*Fagetalia sylvaticae* order). The occurrence of *C. calceolus* was less frequently documented from mixed coniferous communities: *Querco-Pinetum* and *Serratulo-Pinetum* (*Dicrano-Pinion* alliance) [14,71]. Rarely, *C. calceolus* can be observed in thickets and shrub communities developed on calcareous substrates, e.g., hazel or beech bushes, assorted with various tree and shrub species. It also grows in thickets and thermophilous saum communities [44]. To date, only few shrub communities with *C. calceolus* have been identified, like: *Peucedano cervariae-Coryletum* (*Potentillo albae-Quercion* alliance) and the community with *Eupatorium cannabinum* (*Atropion belladonnae* alliance). Occurrence of the species in the communities of *Trifolio-Geranietea sanguinei* class was also mentioned, but not fully described [44].

*Cypripedium calceolus* rarely occurs in areas with high sun exposure, as xerothermic grasslands [34,72] and calcareous high mountain grasslands, where it creates sometimes stable populations. It is noteworthy that *C. calceolus* may be accompanied there by other rare orchids, like: *Epipactis atrorubens*, *Epipogium aphyllum*, *Gymnadenia odoratissima*, *Corallorhiza trifida* and *Orchis mascula*. So far, only two grassland communities with occurrence of *C. calceolus* have been specified: *Onobrychido-Brometum* (*Bromion erecti* alliance) and *Carici sempervirentis-Festucetum tatrae* (*Seslerion tatrae* alliance) [34].

### Appendix A.7. Romania

*Cypripedium calceolus* is considered a rare species in Romania. In the past in Romania *C. calceolus* was reported in many sites in almost 100 localities and most of them located from the lowland to the high mountain region. In 1972, the main flora of Romania at that time mentioned 98 localities of *C. calceolus* [73].

In recent years the populations of *Cypripedium calceolus* have been greatly restrained. The often situations encountered in the recent years are the fact that some of the locations cited in the Romanian flora were repeatedly revisited but the plants were not found again and if the population still exists it is represented by only a few plants or getting smaller each year with only few flowering specimens [37,74,75]. However, in the “Synthetic report concerning the state of conservation of species and habits of community interest from Romania” the general assessment of the state of conservation for *C. calceolus* is considered favorable with unknown tendency [76]. *C. calceolus* is cited as a criterion species for the declaration of Natura 2000 sites and, according to Article 17 of the Council Directive 92/43/EEC, the Government must periodically report its conservation status to the European Environmental Agency. The report was sent by Romania for the period 2007–2012 and submit the presence and the conservation status of *C. calceolus* in the Natura 2000 Network in a 10 × 10 km grid cells. The conservation status was assessed as favorable by our country [76].

European University Information Systems (EUNIS) data for Romania shows 21 Natura 2000 sites with *C. calceolus*, just four sites located in the lower lands and most of them located in the upland regions.

As note of hope for so many lost locations some recent publications have been confirmed the presence of *C. calceolus* in another lower land areas that are not mentioned in the EUNIS database. Two populations of *C. calceolus* have beeninvestigated in the central area of Romania, near Sovata (Mures county) in Ursu Lake Reservation and Sărături Arboretum near Sovata at an altitude of 530 m. The ecocenotic environment for the species is a phytocenoses of the *Carpino-Fagetum* association. Other characteristic species for this area are: *Acer campestre*, *Rubus hirtus*, *Sambucus nigra*, *Sanicula europaea*, *Lathyrus hallersteinii*, *Neottia nidus-avis*, *Lathyrus vernus*, *Festuca heterophylla*, *Galium schultesii*, *Ranunculus auricomus*, *Brachypodium sylvaticum*, *Pulmonaria officinalis*, *Galium odoratum*, *Salvia glutinosa*, *Actaea spicata*, *Mycelis muralis*, *Athyrium filix-femina*, *Anemone nemorosa* [37].

In 2016 in the northern part of Romania in a Natura 2000 site, Făgetul Clujului-Valea Morii (Cluj county), after more than 80 years since the last record in this region a new population of *C. calceolus* was discovered. One population is located at the edge of a small forest opening between *Galio-Carpinetum* oak-hornbeam and Dacian oak-hornbeam forests and another along the *Asperulo-Fagetum* beech forest, a transition area between the forest and a dry calcareous grassland habitat at 486–500 m altitude. The average of tree age is 55–60 years. The floristic composition communities identified are: *Carpino-Fagetum*, *Lathyro hallersteinii-Carpinetum Coldea* and *Carici montanae-Quercetum petraeae*. Other characteristic species for this area are: *Aposeris foetida*, *Lathyrus hallersteinii*, *Carex montana*, *Pulmonaria mollis*, *Carex pillosa*, *Galium schultesii*, *Festuca drymeia*, *Hepatica nobilis*, *Sanicula europaea*, *Cephalanthera longifolia*, *Salvia glutinosa*, *Gymnadenia conopsea*, *Staphylea pinnata*, *Genista tinctoria* etc. [74].

In 2017 in Tudora Reservation (Botosani county), with an altitudinal range from 314 to 513 m, a population of *C. calceolus* was confirmed in an association belonging to the *Geranio robertianae*‒*Fagetum taxetosum baccatae*, Dacian Beech forests (*Symphyto-Fagion*). Other northern populations are mentioned near Suceava (Neamt county) are in a habitat consisted by mixed wood forest of beech and oak. The vegetal associations are *Geranio robertianae-Fagetum* and *Galio schultesii-Fagetum* at 390-410 m altitude [75].

Data regarding habitat and social communities where *C. calceolus* is reported from upland areas are from more open land in mountain hay meadows. One investigated population is located in the Piatra Craiului Mountains (Rucăr village area) in a meadow within a mesophilic pasture near the forest areas characterized by spruce (*Picea abies*). The vegetal association belongs to *Anthoxantho-Agrostetum capillare*, characteristic classification of NATURA 2000 habitat 6520 mountain hay meadows. In this location *C. calceolus* is accompanied by *Trollius europaeus*, *Fragaria vesca*, *Alchemilla vulgaris*, *Anthyllis vulneraria*, *Trifolium montanum*, *T. repens*, *T. pratense*, *Lotus corniculatus*, *Linum catharticum*, *Carum carvi*, *Astrantia major*, *Laserpitium latifolium*, *Pimpinella saxifraga*, *Heracleum sphondylium*, *Polygala amara*, *Gentiana asclepiadea*, *G. cruciata*, *Pedicularis comosa*, *Rhinanthus rumelicus*, *G. cruciata*, *Thymus balcanus*, *Phyteuma tetramerum*, *Centaurea pseudophrygia*, *Listera ovata*, *Orchis morio*, *Neottia nidus-avis*, *Gymnadenia conopsea*, *Platanthera bifolia*, *Briza media*, *Dactylis glomerata*, *Agrostis capillaris*, *Anthoxanthum odoratum*, *Trisetum flavescens* etc. [77].

### Appendix A.8. Russian Federation

The territory of Russian Federation covers some of the largest portions of the total *Cypripedium calceolus* distribution area in Europe, extending from 42° N to 69° N latitudinally, and longitudinally almost throughout. Ecologically *C. calceolus* occupies a wide range of habitats, depending on climatic zone. It tolerates wide range of insolation, varying from deep shade (5–7%) to full sun; similarly, it tolerates wide range of soil humidity, but permanently grows on basic soils, probably everywhere [60,78]; it is accustomed to low soil pH in the northern part of its distribution area, whereas southwards, this amplitude is possibly wider.

The northernmost population of *C. calceolus* in Russia, which is located in Murmansk Region, occurs in light shade (30–50%), in the podzol, peaty podzol or peaty soil [79]; one of populations in Murmansk Region, which was subjected for a detailed study for years, was located in pine forest with *Betula pubescens*, *Salix caprea*, *Juniperus sibirica*, herb layer consisted of *Cirsium heterophyllum*, *Bartsia alpina*, *Potentilla erecta* and other species [79].

In NE and NW Regions of Russia, where *C. calceolus* may be locally numerous in especially favourable conditions, its habitats are described as ‘spruce, birch, and aspen forests, forested swamps, calcium outcrops’ in the Komi Region [80]; ‘grass-swamp and grassy forests composed with various tree species, afforested eutrophic swamps’ in the Karelia Region [81]; and ‘hill slopes in various extend covered by woody vegetation, river slopes, edges of forests and forest glades, wet boggy forests on carbonate soils, old limestone quarries’ in the Leningrad, Pskov and Novgorod Regions [82]. Thus, in this area ecologically discussed species is rather variable, and the number of its localities in this area is sometimes numerous as well.

If we go further south, the ecology of *C. calceolus* is repeatedly described by a combination of similar habitats, although the density of the populations may reduce. Examples: ‘spruce, spruce-broadleaved and broad-leaved forests in the edges of river valleys and ravines, usually in the places where limestone is situated near to the surface of the ground’ in the Vladimir Region [83]; deciduous, coniferous or coniferous-broadleaved forests, edges of swamps’ in the Kaluga Region [39]).

Near to southern species limit, *C. calceolus* is usually recorded only in the shady forests: ‘dark forests’ in Tambov Region [84]; ‘pine-broad-leaved forests on carbonate soil’ in Samara Region [85]; probably, near to the southern limit, in the areas with more arid climate, this species finds favourable conditions in the narrow amplitude of habitats. In four to five southernmost regions of European Russia (Rostov, Volgograd, Astrakhan and Kalmykia Regions), *C. calceolus* is absent. Its presence in the Voronezh Region is uncertain.

In the eastern part of the country, *C. calceolus* is reported predominantly either from forests or bogs. In the Perm’ Region it was reported from ‘coniferous and deciduous forests, boggy spruce forests, on calcareous slopes’ [86]; in the Bashkortostan Region ‘in forests of different composition (…), on forest edges, at the marginal parts of afforested bogs’ [87]. Thus, here we see generally the same amplitude, with the one possible exception that it less often inhabits places with a higher level of insolation.

Interestingly, the influence by large wild animals was reported as having influence on the distribution and spread of this species. It is widely known that many ephemeral tuberous orchids with a short life cycle depend on soil disturbance. It is remarkable that the same was noted for *C. calceolus* with its long-life cycle: ‘positive influence was noted during the period of increase of the number of boars, who were losing the soil, thus contributing to an increase in successful seed germination’ [38].

### Appendix A.9. Serbia and Montenegro

In Serbia, *Cypripedium calceolus* was found in the community *Arctostaphylo-Piceetum* [88], whereas in Montenegro it occurs in the *Pinus nigra* forests [89].

### Appendix A.10. Slovenia

In Slovenia, *Cypripedium calceolus* occurs mainly in different beech forests communities: *Ostryo-Fagetum*, *Arunco-Fagetum*, *Rhododendro hirsuti-Fagetum*, *Anemono trifoliae-Fagetum*, *Homogyno sylvestris-Fagetum*, *Omphalodo-Fagetum* and *Polysticho lonchitis-Fagetum*, and other communities, such as: *Petasiti paradoxi-Piceetum*, *Brachypodio-Pinetum sylvestris*, *Alno incanae-Pinetum sylvestris*, *Lamio orvalae-Alnetum incanae*, *Aceri-Alnetum incanae*, *Adenostylo glabrae-Piceetum*, *Rhodothamno-Pinetum mugo*, *Amelanchiero ovalis-Pinetum mugo*, *Rhododendro hirsuti-Betuletum carpaticae*, *Rhodothamno-Laricetum*, *Astrantio carniolicae-Adenostyletum glabrae* and *Caricetum ferrugineae* s.l. [39,90,91].

## Appendix B

**Table A1:** The IUCN threat category of *Cypripedium calceolus* for all European countries.

Country	IUCN category	IUCN Criteria	References
Greece	EX	-	[92,93]
Bulgaria	CR	B1ab(v)c+2ab(v); C1+2a(i,ii); D	[94]
Serbia	CR	B2dC2b	[89]
Croatia	EN	A4a, A4d	[24,61,63]
Bosnia and Herzegovina	CR	Not evaluated	[95]
Montenegro	EN	D	[96]
Romania	VU	B2ab(i,ii,iii)	[97,98,99]
Poland	VU	B2ab(i, ii, iii, iv)	[14,26]
France	VU	A4acd	[100,101]
Italy	LC	-	[102,103,104]
Estonia	NT	Not evaluated	[105]
UK	CR	D	[106,107]
Spain	EN	C1	[108,109]
Russia (only for *C. calceolus*)	LC	-	[110]
Crimea	CR	Evaluation B1ab(i,ii,iv,v) + 2ab(i,ii,iv,v); C2a(i,ii); D.	[111,112]
Germany	VU	Evaluation A2a+B2ab(i, ii, iii, iv)	[113]
Czech Republic	VU	C1	[114,115,116]
Slovak Republic	NT	-	[117,118]
Belgium	EX	-	[6]
Luxemburg	EX	-	[6]
Ukraine	EN	Not estimated	[10,28,29]
Switzerland	VU	B2ab(iii)	[119]
Austria	NT	-	[120]
Slovenia	VU	Not estimated	[10,121,122]
Hungary	EN	Not estimated	[123,124]
Moldova	CR	D-	[10,125]
Belarus	VU	Not estimated	[10]
Lithuania	VU	Not estimated	[10,126]
Norway	NT	-	[127]
Latvia	VU	Not estimated	[128,129]
Sweden	LC	-	[130]
Finland	NT	A2ab, B2b(iii)	[131]
Denmark	VU	D2	[132,133]
Liechtenstein	EX	-	[10]
The Netherlands	EX	-	[8]
